# A new species of the genus *Acanthosaura* from Yunnan, China (Squamata, Agamidae)

**DOI:** 10.3897/zookeys.888.38491

**Published:** 2019-11-11

**Authors:** Shuo Liu, Dingqi Rao

**Affiliations:** 1 Kunming Natural History Museum of Zoology, Kunming Institute of Zoology, Chinese Academy of Sciences, 32 Jiaochang Donglu, Kunming, Yunnan 650223, China Kunming Institute of Zoology, Chinese Academy of Sciences Kunming China; 2 Kunming Institute of Zoology, Chinese Academy of Sciences, 32 Jiaochang Donglu, Kunming, Yunnan 650223, China Kunming Institute of Zoology, Chinese Academy of Sciences Kunming China

**Keywords:** *
crucigera*, Dehong, *
lepidogaster*, Tongbiguan

## Abstract

A new species of *Acanthosaura* from Yunnan, China is described based on unique morphometric and meristic external characters and a very distinctive color pattern. The fourteenth species recorded of this genus, *Acanthosaura tongbiguanensis***sp. nov.**, was previously considered *A. lepidogaster* although it more closely resembles *A. crucigera*. It can be separated from all other species of the genus by having different numbers of subdigital lamellae on the fourth finger and toe, and a different shape of the black eye patch. The new species differs genetically from investigated congeners by percentage distance of 14.46% to 23.27% (cytochrome b gene).

## Introduction

The genus *Acanthosaura* (Gray, 1831) includes thirteen currently recognized species: *A. armata* (Hardwicke & Gray, 1827); *A. lepidogaster* (Cuvier, 1829); *A. capra* (Günther, 1861); *A. coronata* (Günther, 1861); *A. crucigera* Boulenger, 1885; *A. nataliae* Orlov et al., 2006; *A. bintangensis* Wood et al., 2009; *A. titiwangsaensis* Wood et al., 2009; *A. cardamomensis* Wood et al., 2010; *A. brachypoda* Ananjeva et al., 2011; *A. phuketensis* Pauwels et al., 2015; *A. murphyi* Nguyen et al., 2018; and *A. phongdienensis* Nguyen et al., 2019. It has a very wide distribution, and phylogenetic studies have shown that the genus was in need of revision as it included several undescribed and cryptic species as revealed by molecular data (Kalyabina-Hauf et al. 2004; Ananjeva et al. 2008). Because at least some of its members are difficult to find and similar in appearance, taxonomic research is incomplete, with many species only recognized recently.

During our field research in Dehong Autonomous Prefecture, Yunnan Province, China, we discovered some lizards that looked superficially like *Acanthosaura lepidogaster*. According to Zhao et al. (1999) and Yang et al. (2008), two species of the genus *Acanthosaura* are distributed in China and only *A. lepidogaster* is found in Yunnan Province. Morphological and molecular data show that this population is clearly distinct from all other named species, and we consequently describe and name it herein.

## Materials and methods

Specimens were collected by hand. Photographs were taken to document color pattern in life prior to euthanasia. Liver tissues were stored in 99% ethanol and lizards were preserved in 75% ethanol. Specimens were deposited at Kunming Natural History Museum of Zoology, Kunming Institute of Zoology, Chinese Academy of Sciences.

Forty-nine meristic and mensural characters were noted for each adult specimen of the type series, but only meristic characters were noted on juvenile specimens (see Table 3). Measurements were taken to the nearest 0.1 mm with a digital caliper. Paired measurements were made on the left side, as was done in the recent revisions in the *Acanthosaura crucigera* species group (Wood et al. 2009, 2010; Ananjeva et al. 2011). Paired meristic characters are given as left/right. The list and methodology of measurements and meristic counts follow Wood et al. (2010) and Pauwels et al. (2015):

**BEP** presence (1) or absence (0) of a black eye patch;

**CS** number of canthus rostralis-supraciliary scales, counted from the nasal scale to the posterior end of the ridge at the posterior margin of the orbit;

**DIAS** length of the diastema, measured from the posterior end of the nuchal crest to the anterior end of the dorsal crest;

**DIASN** number of scales in the vertebral crest scale diastema, counted from the posterior end of the nuchal crest to the anterior end of the dorsal crest;

**DS** maximum length of the largest spine in the dorsal crest, measured from the base to the tip;

**DSL** longest dorsal scale, measured at the base below the dorsal crest;

**ESBO** presence (1) or absence (0) of elliptical scales below the orbit;

**EYE** eye diameter, measured from the posterior to the anterior edge of the eye;

**FI** number of subdigital lamellae on the fourth finger;

**FOREL** forelimb length, measured from axilla to the proximal edge of the palmar region;

**GP** size of gular pouch, scored as absent (0), small (1), medium (2), large (3) or very large (4);

**HD** maximum head height, measured across the parietal region;

**HINDL** hindlimb length, measured from groin to the proximal edge of the plantar region;

**HL** head length, measured from posterior edge of the lower jaw to the tip of the snout;

**HW** head width, maximum head width, the width at the level of the tympanum;

**INFRAL** number of infralabials;

**LKP** presence (1) or absence (0) of light knee patch;

**MH** mental height;

**MW** mental width;

**NCS** number of scales between the fifth canthals;

**ND** presence (1) or absence (0) of a black, diamond shaped, nuchal collar;

**NR** number of scales between the nasal and the rostral;

**NS** number of scales between the nasals;

**NSCSL** number of scales from the fifth canthal to the fifth supralabial;

**NSL** maximum length of the largest spine in the nuchal crest measured from the base to the tip;

**NN** number of nuchal crest spines (in addition to characters abbreviations listed in Pauwels et al. 2015);

**NSSLC** number of scales between the seventh supralabial and the sixth canthal;

**NSSOS** number of scales surrounding the occipital spine;

**NSSPS** number of scales surrounding the postorbital spine (in addition to characters abbreviations listed in Pauwelset al. 2015);

**OF** presence (1) or absence (0) of oblique humeral fold;

**ORBIT** orbit diameter, measured from the posterior to the anterior edge of the orbit;

**OS** length of the occipital spine, measured from the base to the tip;

**PM** number of scales bordering the mental;

**PS** postorbital spine length, measured from the base to the tip of the spine;

**RH** rostral height;

**RS** number of scales bordering the rostral scale;

**RW** rostral width;

**SL** snout length, measured from the anterior edge of the orbit to the tip of the snout;

**SUPRAL** number of supralabials;

**SVL** snout-vent length, measured from the tip of the snout to the tip of the vent;

**TL** tail length, measured from the posterior margin of the vent to the tip of the tail;

**TBW** tail base width, maximum width at tail base;

**TD** tympanum diameter, measured horizontally from the anterior to the posterior border of the tympanum;

**TN** scales absent on tympanum (0) or present (1);

**TO** subdigital lamellae on the fourth toe;

**VENT** number of ventral scales, counted at the midline from the anterior edge of the shoulders to the edge of the vent;

**WNC** maximum width of the spines in the nuchal crest, measured at the base;

**WDS** maximum width of the largest dorsal scale below the dorsal crest, measured at the base;

**YAS** presence (1) or absence (0) of a Y-shaped arrangement of enlarged scales on the snout.

We compared the characters of the new collection with the characters of all currently recognized species of *Acanthosaura* (Pauwels et al. 2015; Nguyen et al. 2018, 2019), see Table 4.

The character DIAS of *Acanthosaura brachypoda* is given both as 4.5 and 1.9 mm in the original description, which is based on a single specimen, so this character is not used here for comparisons. The methodology for taking FOREL and HINDL was insufficiently described in the original description of *A. brachypoda* and thus could not be compared here; CS, NCS, NR, NSCSL, NSSLC and NSSOS were not provided in the original description of *A. brachypoda*; NSSLC, PM, ND, LKP, ESBO and OF were not provided in the original description of *A. murphyi*; SL, ORBIT, WNC, FOREL, HINDL, VENT, OS, NSSOS, CS, RS, NS, NSC, NSCSL,NR, NSSLC, PM, YAS, BEP, ESBO and GP were not provided in the original description of *A. phongdienensis*.

Molecular data were generated for three specimens and all available homologous sequences obtained from GenBank, all new sequences have been deposited in GenBank. According to Ananjeva et al. (2008), the sequences whose GenBank accession numbers are AY572873 to AY572886 belong to *Acanthosaura nataliae*, and the sequences whose GenBank accession numbers are AY572896 to AY572899 belong to *Acanthosaura coronata*. According to Nguyen et al. (2019), it can be inferred that the sequences whose GenBank accession numbers are AY572900, AY572904, AY572905, AY572912 to AY572918, AY572922 and AY572923 probably belong to *Acanthosaura phongdienensis*. According to Pauwels et al. (2015), it can be inferred that the sequences whose GenBank accession numbers are AY572887 and AY572889 to AY572894 probably belong to *Acanthosaura phuketensis*. According to Kalyabina-Hauf et al. (2004) and Ananjeva et al. (2008), the sequences whose GenBank accession numbers are AY572928 to AY572930 belong to some unknown species. Two agamids *Pseudocalotes brevipes* (Werner, 1904) and *Calotes versicolor* (Daudin, 1802) were used as the outgroups based on the results from Kalyabina-Hauf et al. (2004). All the GenBank accession numbers for taxa used in the genetic analysis can be found in Table 1. Total genomic DNA was extracted from liver tissue stored in 99% ethanol. Tissue samples were digested using proteinase K, and subsequently purified following a standard phenol/chloroform isolation and ethanol precipitation (Sambrook et al. 1989). PCR was performed using primers new to this paper TBG–F: ATTCTCGCAATACACTACACAAC and TBG–R: TTTCAAATAATACTTGGGAGGTT. Amplification conditions were as follows: after an initial denaturation at 94 °C for 300 s, 31 cycles followed with a denaturation at 94 °C for 45 s, annealing at 42–45 °C for 45 s, and extension at 70 °C for 120 s; cycle sequencing reactions used a two-step program: 15 cycles followed with denaturation at 94 °C for 45 s, annealing at 47–53 °C for 45 s, extension at 70 °C for 60 s, and 15 cycles of denaturation at 94 °C for 45 s and extension at 60 °C for 60 s (Kalyabina-Hauf et al. 2004). We used a ratio of 0.55 H_2_O: 0.30 ExoI: 0.15 SAP to clean the PCR product (Hanke et al. 1994). Amplified mitochondrial cytochrome b (cytb) fragments were sequenced in both directions using an ABI PRISM 3730 Automated DNA Sequencer (Applied Biosystems) following the manufacturer’s protocol (Nguyen et al. 2019).

**Table 1.** Sequences (cytb) used in this study.

Species	Locality	Voucher no.	GenBank no.
*Acanthosaura armata*	Pulau Pinang, Pinang, Malaysia	PCUM	AY572871
	Pulau Pinang, Pinang, Malaysia	PCUM	AY572872
	No data	NSMT-H4595	AB266452
	No data	No data	NC_014175
*Acanthosaura coronata*	Krong Pa, Gia Lai, Vietnam	ROM31985	AY572896
	Dong Nai, Cat Tien, Dong Nai, Vietnam	ROM42240	AY572897
	Dong Nai, Cat Tien, Dong Nai, Vietnam	ROM37083	AY572898
	Dong Nai, Cat Tien, Dong Nai,Vietnam	ROM42241	AY572899
*Acanthosaura crucigera*	Bago Division, Bago Yoma, Myanmar	CAS206626	AY572888
	Bago Division, Bago Yoma, Myanmar	CAS208426	AY572895
*Acanthosaura lepidogaster*	Chi Linh, Hia Duong, Vietnam	ROM31954	AY572901
	Chi Linh, Hia Duong, Vietnam	ROM31957	AY572902
	Chi Linh, Hia Duong, Vietnam	ROM31960	AY572911
	Chi Linh, Hia Duong, Vietnam	ROM35038	AY572903
	Tam Dao, Vinh Phu, Vietnam	ROM30503	AY572906
	Tam Dao, Vinh Phu, Vietnam	ROM30720	AY572907
	Tam Dao, Vinh Phu, Vietnam	ROM30694	AY572908
	Tam Dao, Vinh Phu, Vietnam	ROM30693	AY572927
	Quang Thanh, Cao Bang, Vietnam	ROM36073	AY572909
	Quang Thanh, Cao Bang, Vietnam	ROM36075	AY572910
	Nakai, Khammouane, Laos	FMNH255488	AY572920
	Nakai, Khammouane, Laos	FMNH255487	AY572921
	Thaphabat, Bolikhamxay, Laos	FMNH255491	AY572919
	Hainan, China	MD001	KR092427
	Sa Pa, Lao Cai, Vietnam	ROM38117	AY572924
	Sa Pa, Lao Cai, Vietnam	ROM38115	AY572925
	Sa Pa, Lao Cai, Vietnam	ROM38116	AY572926
*Acanthosaura nataliae*	Krong Pa, Gia Lai, Vietnam	ROM31983	AY572873
	Krong Pa, Gia Lai, Vietnam	ROM32167	AY572874
	Krong Pa, Gia Lai, Vietnam	ROM32160	AY572875
	Krong Pa, Gia Lai, Vietnam	ROM31984	AY572876
	Krong Pa, Gia Lai, Vietnam	ROM32154	AY572877
	Krong Pa, Gia Lai, Vietnam	ROM32155	AY572878
	Krong Pa, Gia Lai, Vietnam	ROM32160	AY572879
	Tram Lap, Gia Lai, Vietnam	ROM30627	AY572880
	Tram Lap, Gia Lai, Vietnam	ROM30628	AY572881
	Krong Pa, Gia Lai, Vietnam	ROM32161	AY572882
	Krong Pa, Gia Lai, Vietnam	ROM32152	AY572883
	Krong Pa, Gia Lai, Vietnam	ROM32162	AY572884
	Krong Pa, Gia Lai, Vietnam	ROM32143	AY572885
	Krong Pa, Gia Lai, Vietnam	ROM32166	AY572886
Acanthosaura cf. phongdienensis	Khe Moi River, Nghe An, Vietnam	ROM26328	AY572900
	Annam, Vu Quang, Ha Tinh, Vietnam	ZISP20753-1	AY572904
	Annam, Vu Quang, Ha Tinh, Vietnam	ZISP20753-2	AY572905
	Boualapha, Khammouane, Laos	FMNH255481	AY572912
	Con Cuong, Nghe An, Vietnam	FMNH255582	AY572913
	Con Cuong, Nghe An, Vietnam	FMNH255583	AY572914
	Tuong Duong, Nghe An, Vietnam	FMNH255585	AY572915
	Tuong Duong, Nghe An, Vietnam	FMNH255587	AY572916
	Con Cuong, Nghe An, Vietnam	FMNH255581	AY572917
	Tuong Duong, Nghe An, Vietnam	FMNH255584	AY572918
	Vieng Tong, Huaphan, Laos	FMNH255489	AY572922
	Khao Yoi, Thailand	PCUM	AY572923
Acanthosaura cf. phuketensis	Kao Yoi, Phetchaburi, Thailand	No data	AY572887
	Khao Lak, TakuaPa, Phang Nga, Thailand	PCUM	AY572889
	Khao Lak, TakuaPa, Phang Nga, Thailand	PCUM	AY572890
	Khao Lak, TakuaPa, Phang Nga, Thailand	PCUM	AY572891
	ThaiMuang, Phang Nga, Thailand	IRSNB15141	AY572892
	Malaysia	No data	AY572893
	Malaysia	No data	AY572894
*Acanthosaura* sp. 1	Myanmar	HLMD-RA2969	AY572929
*Acanthosaura* sp. 1	Myanmar	HLMD-RA2970	AY572930
*Acanthosaura* sp. 2	Ngoc Linh, Kon Tum, Vietnam	ROM37082	AY572928
*Calotes versicolor*	Vietnam	HLDM57	AY572870
*Pseudocalotes brevipes*	Pac Ban, Tuyen Quang, Vietnam	ROM30515	AY572869
*Acanthosaura tongbiguanensis* sp. nov.	Tongbiguan, Dehong, Yunnan, China	KIZL201801	MN604012
	Tongbiguan, Dehong, Yunnan, China	KIZL201802	MN604013
	Tongbiguan, Dehong, Yunnan, China	KIZL201803	MN604014

Sequences were aligned using CLUSTAL X v1.83 (Thompson et al. 1997) with the default parameters and the alignment revised by eye. Pairwise distances between species were calculated in MEGA 7 (Tamura et al. 2011). The best substitution model HKY+G+I was selected using the Akaike Information Criterion (AIC) in MODELTEST v3.7 (Posada and Crandall 1998). Bayesian phylogenetic inference was performed in MRBAYES 3.2.6 (Wang et al. 2009, Ronquist et al. 2012) based on the selected substitution model. Two runs were performed simultaneously with four Markov chains starting from random tree. The chains were run for 1,000,000 generations and sampled every 100 generations. The first 25% of the sampled trees was discarded as burn-in after the standard deviation of split frequencies of the two runs was less than a value of 0.01, and then the remaining trees were used to create a 50% majority-rule consensus tree and to estimate Bayesian posterior probabilities (BPPs). Maximum likelihood analysis was performed in MEGA 7 (Tamura et al. 2011), nodal support was estimated by 1,000 rapid bootstrap replicates.

## Results

**Figure 1.**
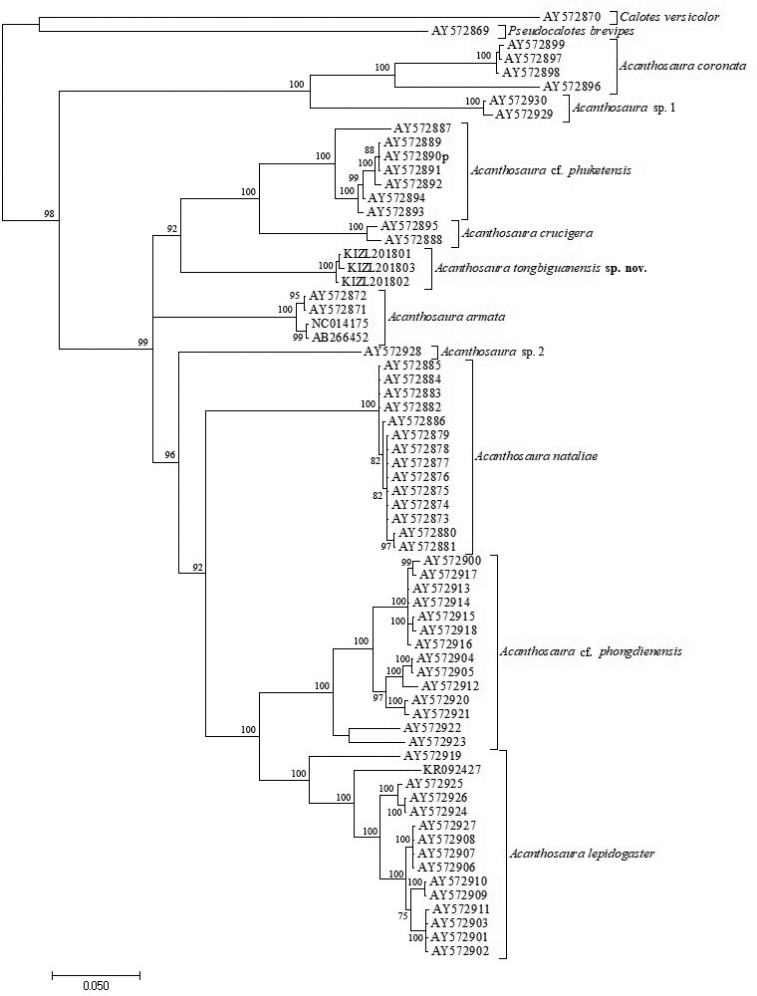
Bayesian phylogram of investigated members of *Acanthosaura* inferred from cytb gene. The nodal numbers are Bayesian posterior probabilities (only values above 70% are shown).

**Figure 2.**
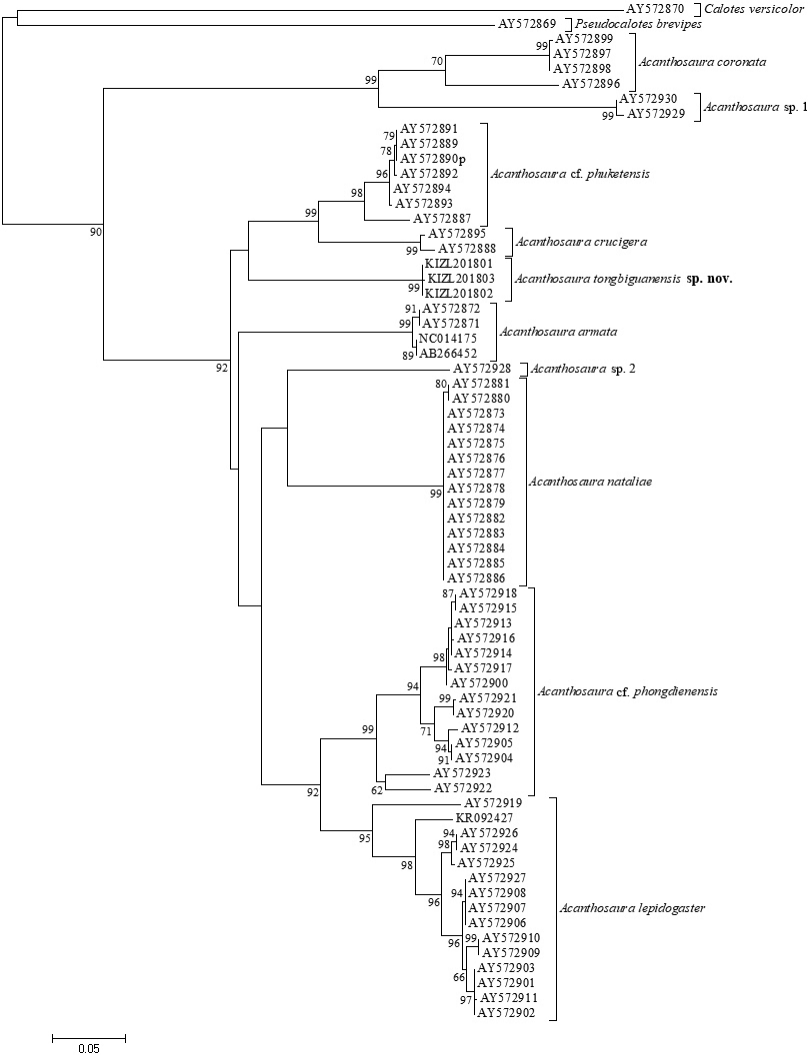
Maximum likelihood phylogram of investigated members of *Acanthosaura* inferred from cytb gene. The nodal numbers are ML bootstrap values (only values above 50% are shown).

The obtained sequence alignment is 795 bp in length. Both Bayesian inference and Maximum likelihood analyses recovered this lineage of the new samples as the sister to the clade consisting of *Acanthosaura crucigera* and A. cf. phuketensis with weak support (Figures 1, 2). The average uncorrected pairwise distances (p-distance) between other investigated members of *Acanthosaura* ranged from 11.17% to 23.9%, the average uncorrected pairwise distances (*p*-distance) between the new species and investigated congeners ranged from 14.46% to 23.27% (Table 2).

**Table 2.** Average uncorrected *p*-distances (%) between investigated members of *Acanthosaura* and outgroups calculated from cytb gene sequences.

**Species**	**1**	**2**	**3**	**4**	**5**	**6**	**7**	**8**	**9**	**10**	**11**
1 *Acanthosaura tongbiguanensis* sp. nov.											
2 *A. armata*	14.80										
3 *A. coronata*	23.27	22.20									
4 *A. crucigera*	15.52	15.11	22.71								
5 *A. lepidogaster*	15.18	16.11	22.48	16.52							
6 *A. nataliae*	14.94	14.90	22.15	15.45	13.72						
7 A. cf. phongdienensis	15.88	14.43	22.63	15.23	12.32	14.77					
8 A. cf. phuketensis	14.46	14.83	22.65	11.17	15.35	15.02	14.04				
9 *Acanthosaura* sp. 1	22.94	21.81	16.18	22.51	23.04	23.77	23.62	23.86			
10 *Acanthosaura* sp. 2	15.35	14.08	23.90	16.47	14.82	15.82	14.93	15.79	23.78		
11 *Pseudocalotes brevipes*	22.85	24.43	26.92	24.32	23.66	24.00	24.79	24.44	26.39	24.67	
12 *Calotes versicolor*	27.25	25.49	28.40	27.60	27.75	27.11	26.39	26.38	28.56	29.33	28.00

### Systematics

#### Acanthosaura tongbiguanensis sp. nov.

Taxon classificationAnimaliaSquamataAgamidae

9927F7C3-7913-5D3F-86F5-DC7B247FB931

http://zoobank.org/6E91FE2B-A4E7-4A7E-BD68-A9C883760883

**Figure 3.**
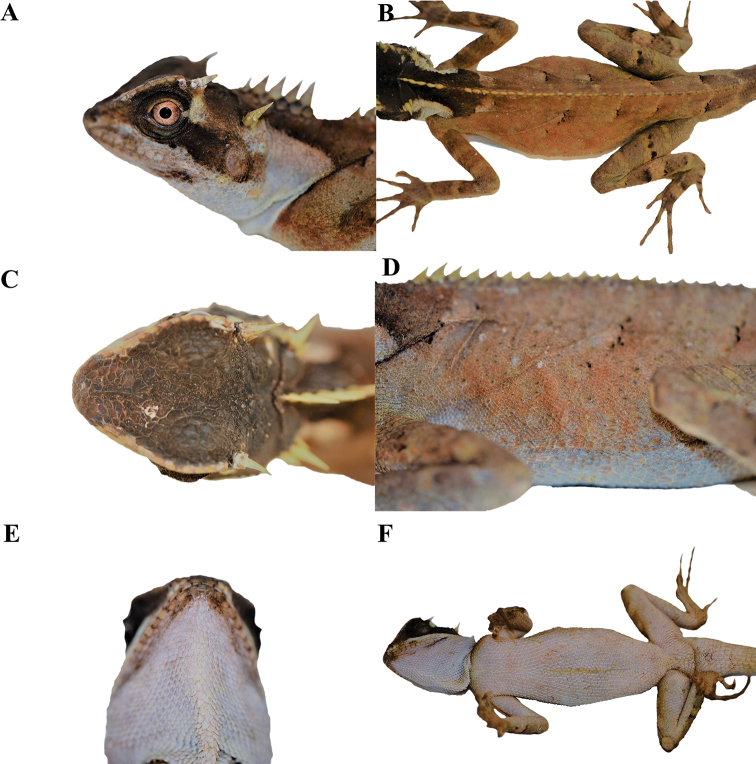
Adult male holotype (KIZL201804) of *Acanthosaura tongbiguanensis* sp. nov. in life **A** lateral view of the head **B** dorsal view of the body **C** dorsal view of the head **D** lateral view of the body **E** ventral view of the head **F** ventral view of the body.

**Figure 4.**
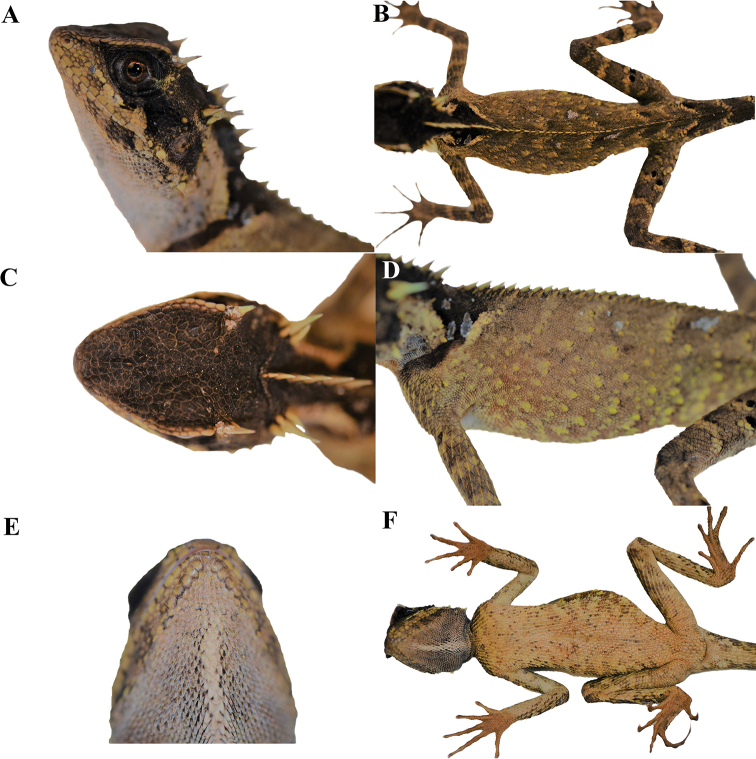
Adult female paratype (KIZL201805) of *Acanthosaura tongbiguanensis* sp. nov. in life **A** lateral view of the head **B** dorsal view of the body **C** dorsal view of the head **D** lateral view of the body **E** ventral view of the head **F** ventral view of the body.

**Figure 5.**
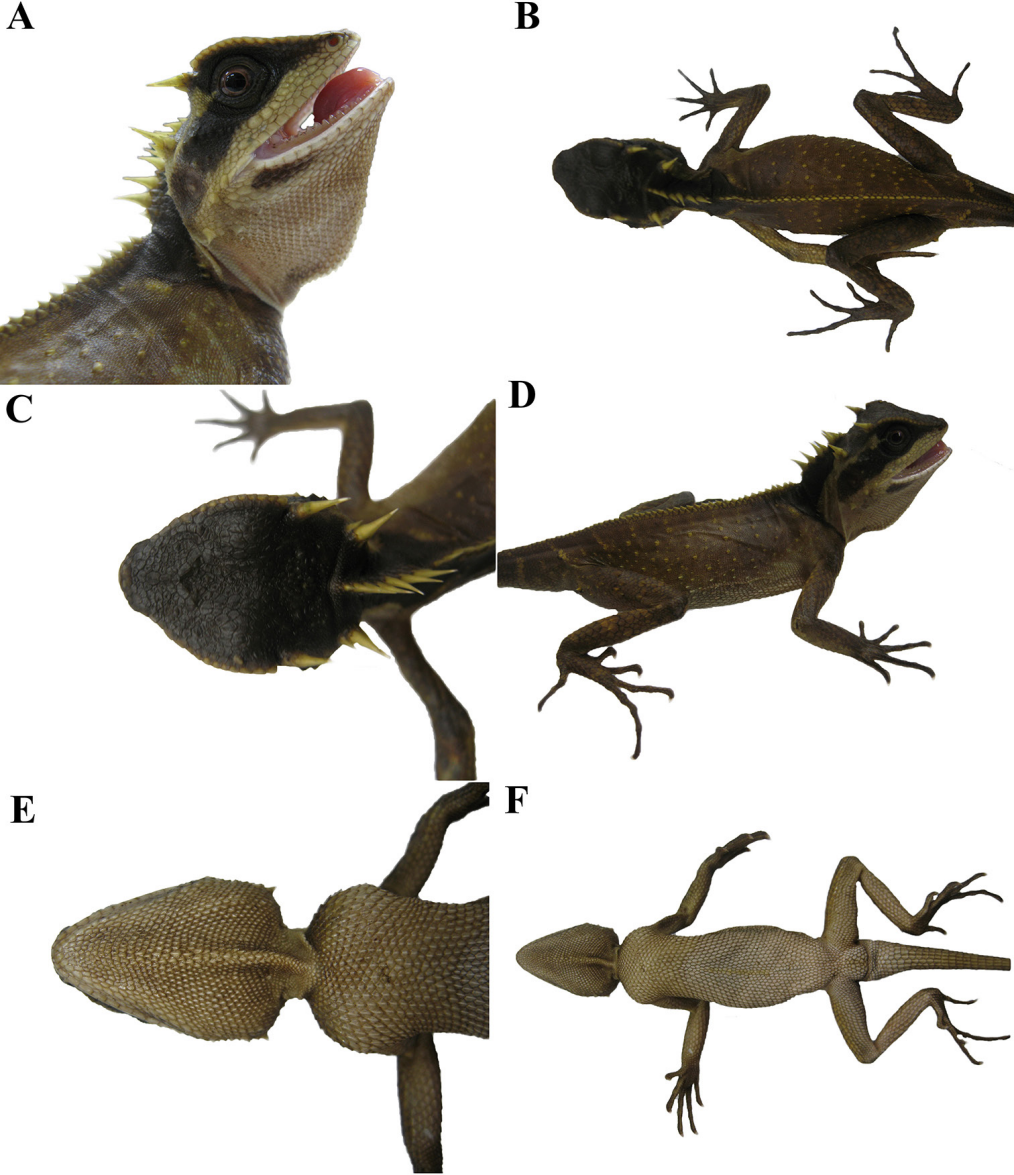
Adult male paratype (KIZL201801) of *Acanthosaura tongbiguanensis* sp. nov. in life **A** lateral view of the head **B** dorsal view of the body **C** dorsal view of the head **D** lateral view of the body **E** ventral view of the head **F** ventral view of the body.

**Figure 6.**
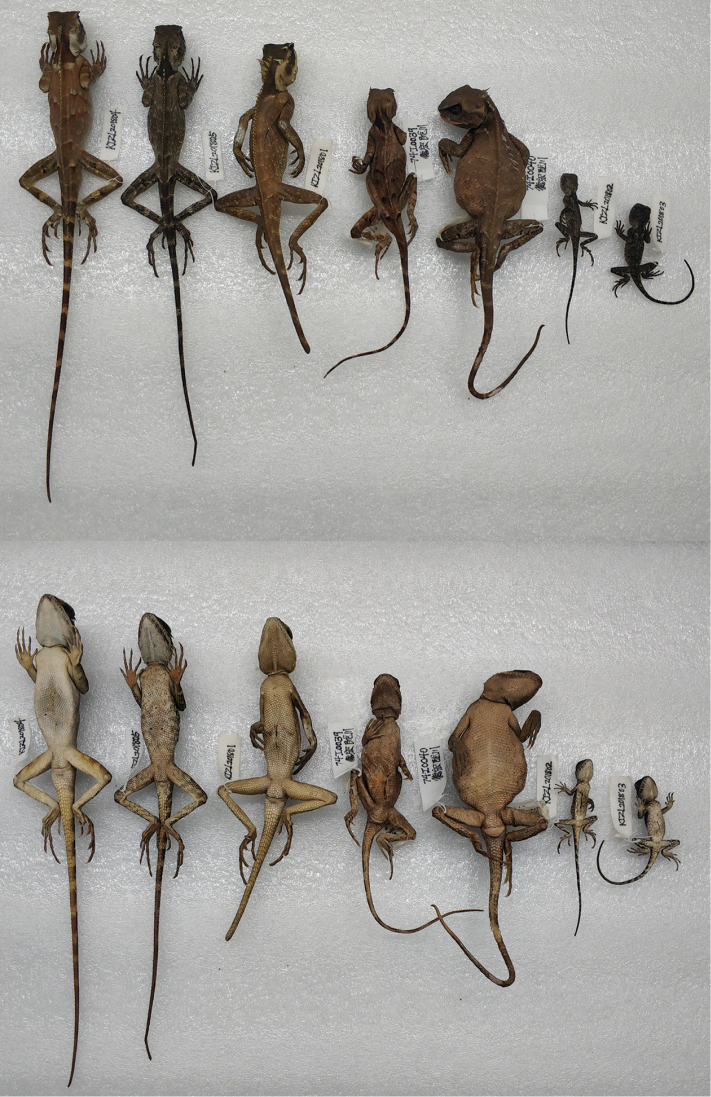
Dorsal view (top) and ventral view (bottom) of type series of *Acanthosaura tongbiguanensis* sp. nov. in preservative. From left to right: male holotype (KIZL201804), female paratype (KIZL201805), male paratype (KIZL201801), female paratype (74I0039), female paratype (74I0040), juvenile paratype (KIZL201802), juvenile paratype (KIZL201803).

Figures 3 , 4 , 5 , 6 , 10

Acanthosaura lepidogaster : Zhao et al. 1999: 82–85.
Acanthosaura lepidogaster : Yang and Rao 2008: 186–187.

##### Type material.

***Holotype*.** KIZL201804, an adult male, 22:18 02 Sept 2018, leg. Shuo Liu, Tongbiguan Township (24°36’51.24”N, 97°35’1.88”E, 1170.24 m elevation), Yingjiang County, Dehong Autonomous Prefecture, Yunnan, China.

***Paratypes*.** KIZL201801, an adult male, 22:53 01 Sept 2018, leg. Shuo Liu, same locality as holotype; KIZL201802 and KIZL201803, two juveniles, 21:00–22:00 02 Sept 2018, leg. Shuo Liu, same locality as holotype; KIZL201805, adult female, 22:40 02 Sept 2018, leg. Shuo Liu, same locality as holotype; 74I0039 and 74I0040, two gravid females, old specimens in the specimen collection room of Kunming Institute of Zoology, Chinese Academy of Sciences, Aug 1974, leg. Longchuan County, Dehong Autonomous Prefecture, Yunnan, China.

##### Etymology.

The name refers to Tongbiguan Nature Reserve, the locality where the new species was found.

##### Diagnosis.

A medium-sized (maximum SVL 115.6 mm) agamid lizard with two pairs of spines: postorbital (supraciliary) spines and spines on occiput between tympanum and nuchal crest; tympanum naked; moderately developed gular pouch; scales on flanks randomly intermixed with medium and large scales; nuchal crest present and strongly developed; diastema between the nuchal and dorsal crests present; dorsal crest slightly developed, composed of enlarged, pointed scales beginning at shoulder region and decreasing regularly in size; tail 1.56–1.85 times SVL; black nuchal collar present; black eye patch present; black oblique folds anterior to the fore limb insertions present.

The new species can be separated from all congeners by having different numbers of subdigital lamellae on the fourth finger (19–21) and toe (25–28), and a different shape of the black eye patch, that extends from posterior margin of nostrils through orbit posteriorly and downwards beyond the posterior end of the tympanum but neither meeting the diamond shaped black nuchal collar on nape nor black oblique humeral fold.

##### Description of the holotype.

Adult male. SVL 110.8 mm. TL 205.0 mm, tail complete. Head length 31.1 mm; head moderately long (HL/SVL 28%), somewhat narrow (HW/SVL 18%), not tall (HD/HL 52%), triangular in dorsal and lateral profile. Snout short (SL/HL 31%); interorbital and frontal regions and rostrum wide, steeply sloping anteriorly. Canthus rostralis prominent, forming a large projecting ridge extending above eye, composed of 11/13 enlarged scales; the ridge terminates with a notch anterior to the postorbital spine. Rostral moderate in size, rectangular; nasal concave, nostrils surrounded by a circular scale. Eye relatively large (EYE/HL 22%), orbit very large (ORBIT/HL 35%). Prefrontal and frontal scales slightly keeled and larger than scales between supralabials; scales on occiput weakly keeled. Moderately elongate epidermal spine above posterior margin of eye, straight, surrounded by 5/4 enlarged scales. A notch present between the supraciliary edge and postorbital spine. Moderately elongate epidermal spine on occipital region, straight, surrounded by a rosette of 5/4 short spiny scales. Tympanum exposed, oblong, surrounded by small scales. Supralabials 13/13, rectangular, scales in center of series largest; mental squarish above, becoming triangular below, larger than first pair of INFRAL; five scales contacting the mental; infralabials 13/12, rectangular, scales in center of series largest; gulars sharply keeled and spinose. Dewlap extensible, gular pouch moderate. Nuchal crest composed of four very elongate, lanceolate, laterally compressed scales and one moderately elongate, lanceolate, laterally compressed scale bordered on each side by one row of enlarged, spinose scales; nuchal crest followed by a diastema at base of nape. Dorsal body crest slightly developed, extending from posterior margin of diastema onto base of tail; vertebral crest composed of enlarged, epidermal, laterally compressed, spinose scales, bordered by a single row of smaller paravertebral spinose scales; vertebral crest tapers slightly to base of tail, then fades progressively. Body slightly short, triangular in cross-section. Dorsal scales small, mixed with large scales indistinctly arranged in slanted forward and downward rows from the midline of the back, keels projecting posterior wards; scales of pectoral region and abdomen larger than dorsal scales, keeled, more or less arranged in transverse rows; keeled scales anterior to vent not enlarged. Limbs relatively long (FOREL/SVL 39%, HINDL/SVL 56%); dorsal and ventral scales of forelimbs keeled, spinose, about the same size. Five digits on manus; subdigital scales keeled, subdigital lamellae under fourth finger 20/21. Scales of hind limbs keeled and spinose; postfemoral scales small, interspersed with larger spinose scales. Five digits on pes; subdigital scales keeled, subdigital lamellae under fourth toe 26/27. Tail length 1.85 times SVL, tail covered with keeled spinose scales, keels on subcaudals directed posteriorly; subcaudals much longer than supracaudals; base of tail 13.1 mm wide.

**Figure 7.**
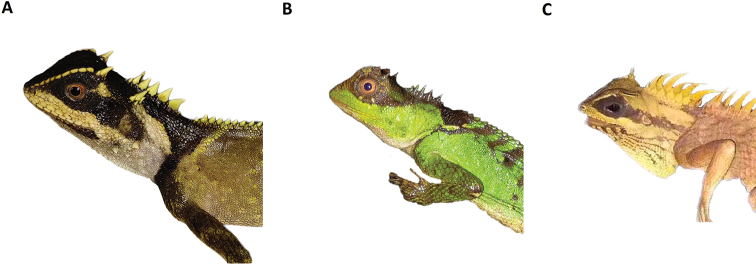
Comparison of three different types of eye patch **A***Acanthosaura tongbiguanensis* sp. nov. **B***A. lepidogaster***C***A. nataliae*.

**Figure 8.**
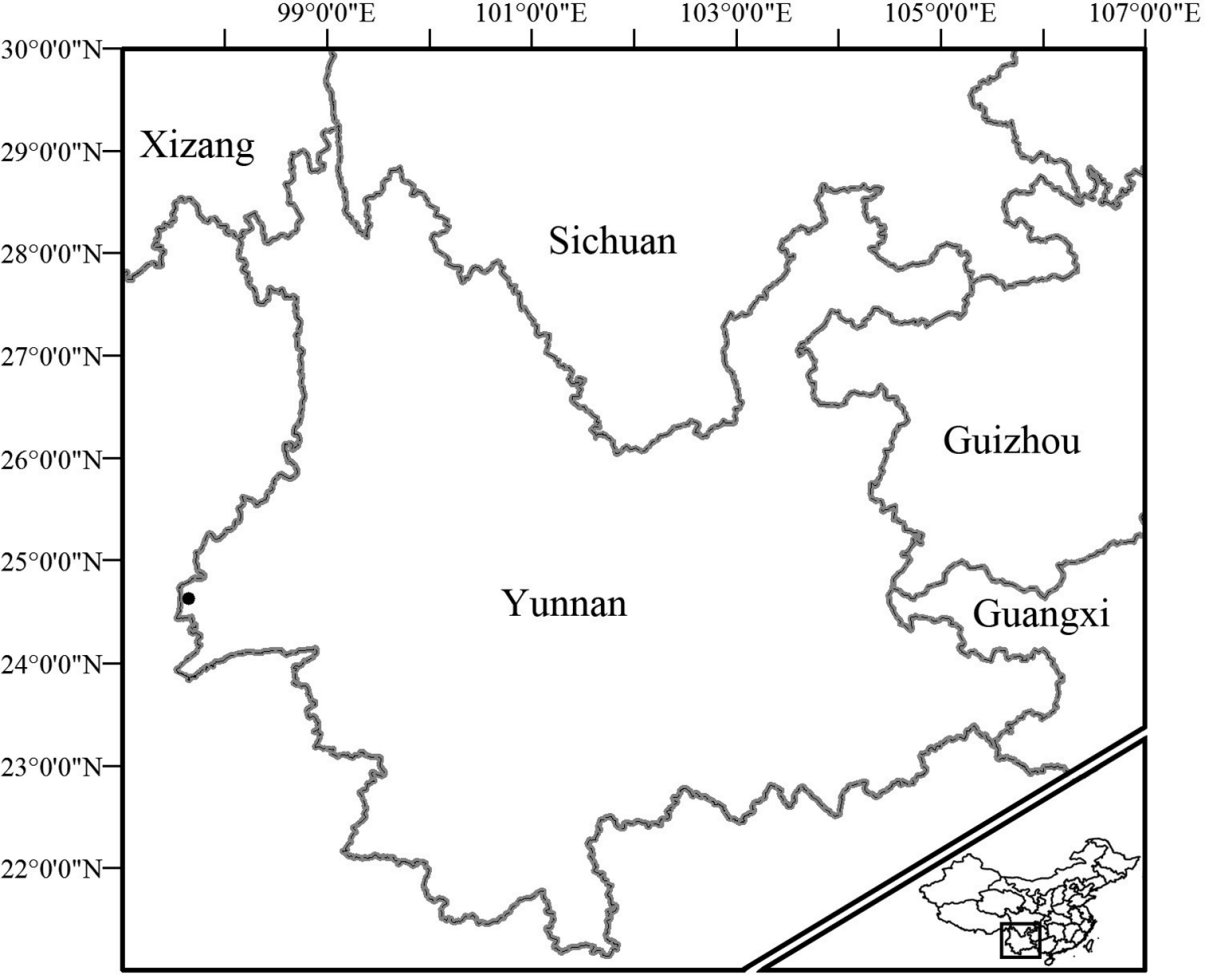
The type locality of *Acanthosaura tongbiguanensis* sp. nov. (black dot) close to the border with Myanmar.

**Figure 9.**
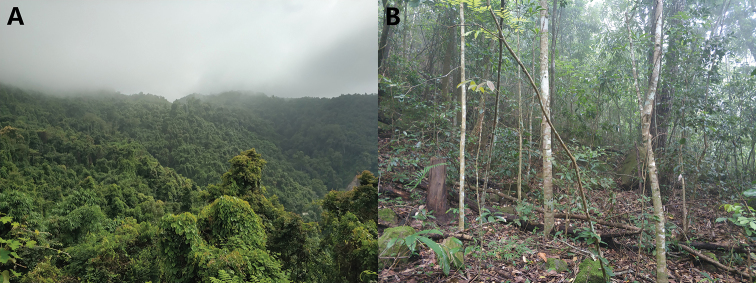
Habitat at the type locality of *Acanthosaura tongbiguanensis* sp. nov., Tongbiguan Township, Yingjiang County, Dehong Autonomous Prefecture, Yunnan, China **A** distant view **B** close view.

##### Color of holotype in life.

Dorsal surface of head black, dorsal surface of body and limbs orangish brown; black eye patch extending from posterior margin of nostrils through orbit posteriorly and downwards beyond the posterior end of the tympanum but neither meeting the diamond shaped black nuchal collar on nape nor black oblique folds anterior to the fore limb insertions; upper lip white, same as color of lateral and ventral sides of neck, lower lip white with small black speckle at posterior region; iris orangish brown; black nuchal collar extending downward to reach black oblique folds anterior to fore limb insertions, two white patches at lower back of black nuchal collar; gular region white; postorbital spines, occipital spines, nuchal crest spines and ridge of the rostral and orbit cream-colored; tongue and inside of mouth pink; few small black speckles and yellow diagonal stripes from midline of the back, irregular light colored spots on sides of body not obvious; stripes checkered with black and white on dorsal ground of limbs and tail; ventral sides of limbs and body white, front part white and back part dark on ventral side of tail. However, it should be noted that this species can change the color of its body within a certain range like most other members of the genus.

##### Variations.

Morphometric and meristic data for the type series are provided in Table 3. The paratypes resemble the holotype in most aspects except that the male KIZL201801 has a darker dorsal ground of the body with no black speckles in the dorsal pattern, and the number of nuchal crest scales is six. The female KIZL201805 has a much darker dorsal ground of the body and irregular black patterns on the ventral sides of the body, limbs, and tail; light colored spots on the sides of the body are more obvious, the color in the gular region is a bit darker. The juveniles KIZL201802 and KIZL201803 have much shorter postorbital, occipital, and nuchal crest spines, and obvious radial patterns around the eyes; the colors of the bodies are relatively darker, the yellow diagonal stripes from the midline of the back are more obvious; they also have irregular black patterns on the ventral sides of the body, limbs, and tail; nuchal crest scales of KIZL201803 numbers four. The females 74II0039 and 74I0040 were not observed alive but only in preservative: the female 74II0039 has a much more obvious black speckling in the dorsal pattern while the female 74II0039 has no black speckles, but they both have irregular black patterns on the ventral sides of the body, limbs, and tail; both have six nuchal crest scales.

**Table 3.** Morphometric (in mm) and meristic data for the type series of *Acanthosaura tongbiguanensis* sp. nov. For character abbreviations see material and methods. Paired meristic characters are given left/right. NA = not applicable.

	Adult males		Adult females			Juveniles		Range	Mean
	Holotype	Paratype	Paratypes			Paratypes			
	KIZL201804	KIZL201801	KIZL201805	74II0039	74I0040	KIZL201802	KIZL201803		
SVL	110.8	108.1	107.2	93.0	115.6	NA	NA	93.0–115.6	106.9
TL	205.0	NA	180.0	144.9	183.5	NA	NA	144.9–205.0	178.4
TL/SVL	1.85	NA	1.68	1.56	1.59	NA	NA	1.56–1.85	1.7
TBW	13.1	13.0	9.2	10.5	12.1	NA	NA	9.2–13.1	11.6
HL	31.1	33.0	30.4	27.5	33.2	NA	NA	27.5–33.2	31.0
HW	20.0	22.0	18.8	18.6	23.3	NA	NA	18.6–23.3	20.5
HD	16.1	17.4	15.7	13.9	17.3	NA	NA	13.9–17.4	16.1
SL	9.8	10.3	10.1	9.2	11.0	NA	NA	9.2–11.0	10.1
ORBIT	11.0	10.6	10.2	7.7	10.6	NA	NA	7.7–11.0	10.0
EYE	6.9	5.8	6.9	6.0	7.3	NA	NA	5.8–7.3	6.6
TD	3.6	4.2	3.3	3.2	3.7	NA	NA	3.2–4.2	3.6
TD/HD	0.22	0.24	0.21	0.23	0.21	NA	NA	0.21–0.24	0.2
TN	0	0	0	0	0	0	0	0	0
PS	5.3	6.3	4.6	3.6	5.4	NA	NA	3.6–6.3	5.0
PS/HL	0.17	0.19	0.15	0.13	0.16	NA	NA	0.13–0.19	0.2
NSSPS	5/4	5/5	5/5	5/5	5/NA	4/4	4/4	4/4–5/5	4.7/4.5
NSL	6.5	6.7	5.4	4.0	6.2	NA	NA	4.0–6.7	5.8
NSL/HL	0.21	0.20	0.18	0.15	0.19	NA	NA	0.15–0.21	0.2
DS	3.7	4.2	3.7	2.4	3.8	NA	NA	2.4–4.2	3.6
DS/HL	0.12	0.13	0.12	0.09	0.11	NA	NA	0.09–0.13	0.1
NN	5	6	5	6	6	5	4	4–6	5.3
DSL	1.9	1.9	1.8	1.7	2.6	NA	NA	1.7–2.6	2.0
WNC	1.2	1.5	1.0	1.1	1.5	NA	NA	1.0–1.5	1.3
WDS	1.4	1.5	1.5	1.4	2.1	NA	NA	1.4–2.1	1.6
DIAS	6.0	5.6	5.5	6.1	3.9	NA	NA	3.9–6.1	5.4
DIAS/SVL	0.05	0.05	0.05	0.07	0.03	NA	NA	0.03–0.07	0.1
DIASN	10	8	9	8	6	7	9	6–10	8.1
FOREL	43.2	42.5	42.4	34.7	41.3	NA	NA	34.7–43.2	40.8
HINDL	62.1	63.9	62.5	54.1	62.9	NA	NA	54.1–63.9	61.1
SUPRAL	13/13	14/14	13/13	11/11	13/13	11/11	12/12	11–14	12.4/12.4
INFRAL	13/12	13/14	14/14	12/11	12/12	10/11	12/13	10–14	12.3/12.4
VENT	66	59	62	52	53	53	60	52–66	57.9
FI	20/21	20/19	21/20	19/19	21/20	20/20	20/21	19–21	20.1/20.0
TO	26/27	25/26	28/27	27/26	26/25	26/27	25/26	25–28	26.1/26.3
OS	6.1	7.0	6.9	4.5	6.3	NA	NA	4.5–7.0	6.2
OS/HL	0.20	0.21	0.23	0.16	0.19	NA	NA	0.16–0.23	0.2
NSSOS	5/4	5/5	4/4	5/5	5/5	5/5	4/4	4–5	4.7/4.6
CS	11/13	14/15	13/13	10/10	12/11	11/10	13/11	10–14	12.0/11.9
RW	3.7	4.5	3.4	3.3	3.5	NA	NA	3.3–4.5	3.7
RH	1.6	1.7	2.0	1.0	1.9	NA	NA	1.0–2.0	1.6
RS	9	9	7	7	7	6	6	6–9	7.3
NS	9	8	8	8	8	8	8	8–9	8.1
NCS	11	11	10	12	13	11	10	10–13	11.1
NSCSL	9	8	9	7	8	9	8	7–9	8.3
NR	2	2	2	2	2	2	2	2	2
NSSLC	13	10	13	10	9	11	12	9–13	11.1
MW	1.6	1.4	1.4	1.9	1.5	NA	NA	1.4–1.9	1.6
MH	1.2	2.0	1.3	1.4	1.7	NA	NA	1.2–2.0	1.5
PM	5	4	5	5	4	4	5	4–5	4.6
YAS	1	1	1	1	1	1	1	1	1
ND	1	1	1	1	1	1	1	1	1
LKP	1	1	1	1	1	1	1	1	1
BEP	1	1	1	1	1	1	1	1	1
ESBO	0	0	0	0	0	0	0	0	0
GP	2	2	2	2	2	1	1	1–2	1.7
OF	1	1	1	1	1	1	1	1	1

##### Distribution.

*Acanthosaura tongbiguanensis* sp. nov. is only recorded in Tongbiguan Nature Reserve including Yingjiang County, Longchuan County and Ruili City, the border region with northern Myanmar in western Yunnan, China, so it probably occurs in northern Myanmar.

##### Natural history.

The type series of *Acanthosaura tongbiguanensis* sp. nov. was collected at night while they were asleep on small trees in a primordial forest. However, we suppose that they forage for food on the ground during the day. At the type locality we found four other species of reptiles, namely *Cyrtodactylus khasiensis* (Jerdon, 1870), *Pseudocalotes kakhienensis* (Anderson, 1879); *P. microlepis* (Boulenger, 1887); *Trimeresurus yingjiangensis* Chen et al., 2019; and seven species of amphibians, *Leptobrachella yingjiangensis* (Yang et al., 2018); *Limnonectes longchuanensis* Suwannapoom et al., 2016; *Megophrys feii* Yang et al., 2018; *M. glandulosa* Fei et al., 1990; *Raorchestes longchuanensis* (Yang & Li, 1978); *Theloderma moloch* (Annandale, 1912); *Zhangixalus smaragdinus* (Blyth, 1852).

##### Comparisons.

Table 4 shows a comparison of morphometric and meristic data for all currently recognized species of *Acanthosaura* and *Acanthosaura tongbiguanensis* sp. nov. It is based mostly on the interspecific comparison tables provided by Pauwels et al. (2015: table 2), Nguyen et al. (2018: table 3) and Nguyen et al. (2019: table 3).

**Table 4.** Comparisons of morphometric (in mm) and meristic data for all currently recognized species of *Acanthosaura* and *Acanthosaura tongbiguanensis* sp. nov., “?” = data not available. **(1)***Acanthosaura tongbiguanensis* sp. nov.; **(2)***A. armata*; **(3)***A. bintangensis*; **(4)***A. brachypoda*; **(5)***A. capra*; **(6)***A. cardamomensis*; **(7)***A. coronata*; **(8)***A. crucigera*; **(9)***A. lepidogaster*; **(10)***A. murphyi*; **(11)***A. nataliae*; **(12)***A. phongdienensis*; **(13)***A. phuketensis*; **(14)***A. titiwangsaensis*.

	(1)	(2)	(3)	(4)	(5)	(6)	(7)	(8)	(9)	(10)	(11)	(12)	(13)	(14)
SVL	93.0–115.6	72.4–138.0	83.9–142.0	117	94.0–137.9	82–149	66.0–86.1	92.2–127.0	76.5–101.1	103.7–127.3	106.7–158.0	58.5–77.4	69.2–123.5	91.8–118.4
TL	144.9–205.0	96.6–190	112.8–206.0	185.4	133.6–182.1	103–188	86.3–105.0	130.0–174.0	130.6–144.1	159.3–195.8	171.0–287.0	94.6–137.2	107.0–205.6	136.0–174.0
HL	27.5–33.2	6.6–33.7	16.9–25.4	30.3	16.3–38.9	16.3–42.4	14.4–16.3	18.7–23.6	18.9–29.7	29.1–36.8	25.2–43.6	18.6–23.8	19.7–31.4	20.0–24.3
HW	18.6–23.3	15.3–23.0	17.5–23.4	20.6	16.8–27.0	16.4–27.7	13.6–17.5	16.0–22.3	13.4–19.1	20.3–24.6	20.2–27.8	13.1–15.9	14.4–22.8	17.5–23.4
HD	13.9–17.4	12.2–18.9	15.0–19.2	17.2	14.8–24.3	12.6–21.7	11.9–16.8	15.7–22.5	12.0–12.5	18.5–20.6	16.9–24.9	10.4–13.6	10.9–18.6	15.7–20.2
SL	9.2–11.0	6.3–16.6	7.9–11.3	12.2	7.6–16.6	8.6–18.7	6.9–8.4	8.7–12.1	9.3–10.2	10.3–15.3	12.0–19.9	?	6.8–11.0	9.7–12.5
ORBIT	7.7–11.0	5.4–12.2	8.4–12.6	8.3	7.6–11.6	6.0–12.7	6.9–7.5	8.9–10.8	4.7–9.1	9.9–12.3	7.2–10.9	?	6.6–11.2	9.8–13.2
TD	3.2–4.2	2.4–5.2	2.5–3.0	3.6	3.4–5.2	2.5–5.8	1.7–2.8	2.5–3.9	2.2–3.0	3.2–5.2	3.9–7.0	1.78–2.81	3.5–4.7	2.7–4.0
TD/HD	0.21–0.24	0.19–0.28	0.16	0.21	0.21–0.23	0.20–0.27	0.14–0.17	0.14–0.21	0.18–0.24	0.17–0.28	0.23–0.28	0.17–0.22	0.22–0.33	0.17–0.20
TN	0	0	0	0	0	0	0	0	0–1	1	0	0	0	0
PS	3.6–6.3	4.9–9.9	1.9–4.2	3.2	5.2–10.2	3.2–12.7	Absent	1.9–7.8	1.5–2.5	5.6–11.8	7.7–17.8	1.18–2.07	4.6–11.8	3.3–4.4
PS/HL	0.13–0.19	0.22–0.56	0.07–0.19	0.11	0.36	0.14–0.45	0	0.09–0.33	0.06–0.11	0.16–0.34	0.36	0.06–0.09	0.23–0.38	0.14–0.18
NSL	4.0–6.7	5.5–11.2	1.3–4.7	4.7	4.2–14.7	3.8–17.4	0	3.1–8.9	2.9–3.4	7.0–14.9	8.5–23.8	1.24–4.18	4.1–12.2	2.7–4.4
NSL/HL	0.15–0.21	0.22–0.51	0.17–0.21	0.16	0.42–0.43	0.17–0.66	0	0.14–0.38	0.12–0.15	0.24–0.43	0.58	0.07–0.18	0.21–0.39	0.11–0.18
DS	2.4–4.2	4.9–11.3	1.8–2.2	1.9	3.5–6.8	2.0–14.2	Absent	2.0–5.5	1.5–2.7	2.6–10.5	6.0–17.7	0.58–1.65	2.3–8.3	1.7–2.1
DS/HL	0.09–0.13	0.20–0.52	0.08–0.09	0.06	0.16–0.17	0.14–0.45	0	0.09–0.24	0.07–0.12	0.14–0.51	0.44	0.03–0.07	0.11–0.26	0.07–0.09
WNC	1.0–1.5	1.0–2.2	1.6–2.1	1.6	2.3–4.1	1.8–4.2	0	1.3–3.4	1.5	2.9–4.8	3.1–4.8	?	1.4–2.9	1.4–1.6
DIAS	3.9–6.1	1.2–6.8	5.0–7.9	?	2.0–6.7	2.7–8.3	Absent	4.9–8.4	6.3	2.6–4.8	2.5	Absent	3.6–7.6	5.1–7.6
DIASN	6–10	1–8	11–15	7	4–7	6–15	Absent	9–25	10–12	4–8	10	Absent	12–17	10–13
DIAS/SVL	0.03–0.07	0.01–0.06	0.04–0.07	?	0.05	0.03–0.07	Absent	0.04–0.08	0.08	0.02–0.04	0.04	Absent	0.05–0.08	0.05–0.07
FOREL	34.7–43.2	33.7–48.9	33.9–61.5	?	54.2–83.8	31.7–56.8	30.2–35.3	35.6–49.8	33.0–37.1	49.8–56.6	60.0–85.0	?	22.3–42.9	38.0–51.7
HINDL	54.1–63.9	39.0–69.6	43.3–68.6	?	78.5–107.2	42.0–77.1	38.4–47.8	48.8–65.0	49.4–50.4	60.4–68.4	85.0–129.7	?	38.2–60.6	48.5–65.6
SUPRAL	11–14	10–14	12	12–13	10	11–15	12–13	10–13	10–13	12–14	11	9–12	10–12	12–13
INFRAL	10–14	12–15	11–12	11	12–13	10–14	11–13	10–12	9–13	12–14	11–12	10–11	10–12	11–12
VENT	52–66	51–68	51–55	63	55–66	50–65	53–58	55–63	55–61	55–65	64–71	?	57–67	47–57
FI	19–21	13–17	23	18	16–17	15–20	13–14	16–18	17–19	15–18	16–21	14–17	15–17	20–21
TO	25–28	19–26	26–28	24	22–24	20–25	17–19	21–26	22–23	21–23	20–27	19–23	21–24	23–27
TL/SVL	1.56–1.85	1.2–1.6	1.3–1.4	1.58	1.2–1.5	1.2–1.6	0.6–1.0	1.1–1.8	1.6–1.9	1.48–1.54	1.2–1.5	1.5–1.9	1.4–1.7	1.1–1.5
OS	4.5–7.0	4.0–9.4	1.2–2.6	1.0	Absent	4.1–13.3	0	2.5–4.9	3.2–3.4	Absent	Absent	?	2.6–9.5	1.8–2.3
OS/HL	0.16–0.23	0.16–0.38	0.10–0.11	0.03	0	0.24–0.56	0	0.11–0.50	0.14–0.15	0	0	?	0.13–0.30	0.09–0.10
NSSOS	4–5	4–6	6–7	?	Absent	4–6	4–5	4–6	5	Absent	Absent	?	4–5	4–5
CS	10–14	11–15	14–15	?	12–14	11–16	12–15	12–15	10–12	12–14	13	9–13	10–14	14–15
RW	3.3–4.5	1.8–4.5	3.6–5.3	3.5	4.2–4.6	1.7–4.7	0.8–0.9	2.7–4.0	2.8–3.0	3.3–5.1	6.1	2.07–2.65	2.3–3.8	3.6–5.2
RH	1.0–2.0	0.9–1.8	1.7–2.0	2.3	1.8–2.3	1.1–2.2	0.5–0.8	1.3–2.0	1.4–1.5	1.2–2.0	2.6	1.00–1.32	1.1–1.7	1.4–1.8
RS	6–9	5–9	7–9	7	7–8	5–9	5	7–9	5–9	8–9	6	?	5–8	9
NS	8–9	7–10	8	9	9	7–10	7–9	7–9	7–8	7–8	8	?	7–8	8
NCS	10–13	10–17	10–11	?	9	9–17	8–11	9–12	7–10	13–16	14	?	12–13	11–12
NSCSL	7–9	6–14	7–8	?	7–8	7–12	5–6	7–11	8	7–10	8	?	8–10	9–11
NR	2	1–2	1	?	1–2	1–2	3–4	1–2	1–2	3–4	1	?	1–2	1–2
NSSLC	9–13	10–22	9–12	?	9–11	10–19	6–11	10–14	10	?	16	?	11–14	11–14
MW	1.4–1.9	0.9–2.0	1.3–1.8	2.9	1.9–2.2	0.2–2.1	0.6–1.5	1.0–1.5	1.2–1.3	1.7–2.2	2.9	0.87–1.52	0.5–1.4	1.4–2.0
MH	1.2–2.0	0.8–2.2	1.4–2.1	2.1	1.7–2.2	0.9–2.0	1.3–1.6	1.1–1.7	1.2–1.3	1.4–2.0	2.0	1.04–1.60	0.6–1.6	1.4–2.4
PM	4–5	3–6	4–5	4	4	4–5	4–5	4	5	?	4	?	4	5
YAS	1	0–1	1	1	1	0–1	0–1	1	1	0–1	1	?	0–1	1
ND	1	0–1	1	1	1	1	0	1	1	?	0	1	1	1
LKP	1	1	0	1	1	1	1	1	1	?	0	1	1	0
BEP	1	0	1	1	1	1	0	1	0–1	0–1	1	?	1	1
ESBO	0	0	1	0	0	0	0	0	0	?	0	?	0	0
GP	1–2	1	3–4	0	3–4	1–4	0	1–2	0–1	4	4	?	0–2	2–4
OF	1	1	1	1	1	1	1	1	1	?	1	1	1	1

*Acanthosaura tongbiguanensis* sp. nov. can be distinguished from *A. armata* by having more subdigital lamellae on the fourth finger (19–21 vs. 13–17) and the fourth toe (25–28 vs. 19–26), shorter postorbital spines (3.6–6.3 vs. 4.9–9.9 mm, PS/HL 0.13–0.19 vs. 0.22–0.56) and shorter occipital spines (4.5–7.0 vs. 4.0–9.4 mm, OS/HL 0.16–0.23 vs. 0.16–0.38), much shorter nuchal crest spines (4.0–6.7 vs. 5.5–11.2 mm, NSL/HL 0.15–0.21 vs. 0.22–0.51) and much shorter dorsal crest spines (2.4–4.2 vs. 4.9–11.3 mm, DS/HL 0.09–0.13 vs. 0.20–0.52), a higher number of scales in the diastema between the nuchal and the dorsal crests (6–10 vs. 1–8), a relatively longer tail (TL/SVL 1.56–1.85 vs. 1.2–1.6). *Acanthosaura tongbiguanensis* sp. nov. has a black eye patch (vs. absent) and an obvious black nuchal collar (vs. not obvious or absent); *Acanthosaura tongbiguanensis* sp. nov. has fewer or no spots on the dorsal surface of the body, whereas *A. armata* has more spots on the dorsal surface of the body.

The new species can be distinguished from *Acanthosaura bintangensis* by having a larger tympanum (3.2–4.2 vs. 2.5–3.0 mm, TD/HD 0.21–0.24 vs. 0.16), longer head (27.5–33.2 vs. 16.9–25.4 mm), longer postorbital spines (6.3 vs. 4.2 mm), higher maximal length of spines in the nuchal crest (6.7 vs. 4.7), longer spines in the dorsal crest (2.4–4.2 vs. 1.8–2.2 mm, DS/HL 0.09–0.13 vs. 0.08–0.09), less subdigital lamellae on the fourth finger (19–21 vs. 23), much longer occipital spines (4.5–7.0 vs. 1.2–2.6 mm, OS/HL 0.16–0.23 vs. 0.10–0.11), less scales surrounding the occipital spine (4–5 vs. 6–7), lower number of scales in the diastema between the nuchal and the dorsal crests (6–10 vs. 11–15), presence of a light knee patch (vs. absence), less developed gular pouch (1–2 vs. 3–4), absence of an enlarged row of keeled scales below orbit (vs. presence), absence of large yellow spots edged in blackish-brown arranged on body and base of tail (vs. presence); the black eye patch in *Acanthosaura tongbiguanensis* sp. nov. extends backward and downward beyond the posterior end of the tympanum while it never extends onto the head side in *A. bintangensis* (Wood et al. 2009).

From *Acanthosaura brachypoda*, *Acanthosaura tongbiguanensis* sp. nov. can be differentiated by having more subdigital lamellae on the fourth finger (19–21 vs. 18) and the fourth toe (25–28 vs. 24), longer postorbital spines (3.6–6.3 vs. 3.2 mm, PS/HL 0.13–0.19 vs. 0.11) and longer occipital spines (4.5–7.0 vs. 1.0 mm, OS/HL 0.16–0.23 vs. 0.03), much longer spines in the dorsal crest (2.4–4.2 vs. 1.9 mm, DS/HL 0.09–0.13 vs. 0.06), the presence of gular pouch (vs. absence). *Acanthosaura tongbiguanensis* sp. nov. does not have pairs of transverse creamy spots along both sides of spine forming a symmetrical pattern present as in *A. brachypoda*.

*Acanthosaura tongbiguanensis* sp. nov. can be distinguished from *A. capra* based on its smaller body size (93.0–115.6 vs. 94.0–137.9 mm) but longer tail (144.9–205.0 vs. 133.6–182.1 mm, TL/SVL 1.56–1.85 vs. 1.2–1.5), a higher number of subdigital lamellae on the fourth finger (19–21 vs. 16–17) and the fourth toe (25–28 vs. 22–24), lower maximal length of forelimb (43.2 vs. 83.8 mm) and hindlimb (63.9 vs. 107.2 mm), shorter postorbital spines (3.6–6.3 vs. 5.2–10.2 mm, PS/HL 0.13–0.19 vs. 0.36), nuchal crest spines (4.0–6.7 vs. 4.2–14.7 mm, NSL/HL 0.15–0.21 vs. 0.42–0.43) and dorsal crest spines (2.4–4.2 vs. 3.5–6.8 mm, DS/HL 0.09–0.13 vs. 0.16–0.17),lower width of the nuchal crest spines (1.0–1.5 vs. 2.3–4.1 mm), higher number of scales in the diastema between nuchal and dorsal crests (6–10 vs. 4–7), presence of occipital spines (vs. absence), a smaller gular pouch (1–2 vs. 3–4); the black eye patch in *Acanthosaura tongbiguanensis* sp. nov. extends backward and downward beyond the posterior end of the tympanum, while it usually extends backwards and upwards to reach the nuchal crests in *A. capra*.

From *Acanthosaura cardamomensis*, the new species can be separated based on a higher number of subdigital lamellae on the fourth finger (19–21 vs. 15–20) and the fourth toe (25–28 vs. 20–25), a longer tail (144.9–205.0 vs. 103–188 mm, TL/SVL 1.56–1.85 vs. 1.2–1.6), much shorter postorbital spines (3.6–6.3 vs. 3.2–12.7 mm, PS/HL 0.13–0.19 vs. 0.14–0.45), occipital spines (4.5–7.0 vs. 4.1–13.3 mm, OS/HL 0.16–0.23 vs. 0.24–0.56), nuchal crest spines (4.0–6.7 vs. 3.8–17.4 mm, NSL/HL 0.15–0.21 vs. 0.17–0.66) and dorsal crest spines (2.4–4.2 vs. 2.0–14.2 mm, DS/HL 0.09–0.13 vs. 0.14–0.45), a lower width of nuchal crest spines (1.0–1.5 vs. 1.8–4.2 mm); the black eye patch in *Acanthosaura tongbiguanensis* sp. nov. extends backward and downward beyond the posterior end of the tympanum but never reaches the dark nuchal marking on nape while it does so in *A. cardamomensis* (see species’ description and photographs in Wood et al. 2010), besides, *Acanthosaura tongbiguanensis* sp. nov. has fewer or no spots on the dorsal surface of the body, whereas *A. cardamomensis* has more spots on the dorsal surface of the body.

*Acanthosaura tongbiguanensis* sp. nov. is distinguishable from *A. coronata* based on its much bigger body size (93.0–115.6 vs. 66.0–86.1 mm), much longer tail (144.9–205.0 vs. 86.3–105.0 mm, TL/SVL 1.56–1.85 vs. 0.6–1.0), higher number of subdigital lamellae on the fourth finger (19–21 vs. 17–19) and the fourth toe (25–28 vs. 22–23), relatively larger tympanum (TD/HD 0.21–0.24 vs. 0.14–0.17), bigger rostral (RW 3.4–4.5 vs. 0.8–0.9 mm, RH 1.0–2.0 vs. 0.5–0.8 mm), the presence of postorbital spines, occipital spines, nuchal and dorsal crests (vs. absence or not obvious), a diastema between nuchal crest and dorsal crest (vs. a continuous nuchal and dorsal crest), presence of a black nuchal collar (vs. absence), presence of a black eye patch (vs. absence), and the presence of a gular pouch (vs. absence) (see the original description by Günther 1861 and expanded descriptions by Günther 1864; Boulenger 1885).

*Acanthosaura tongbiguanensis* sp. nov. can be differentiated from *A. crucigera* by having more subdigital lamellae on the fourth finger (19–21 vs. 16–18) and the fourth toe (25–28 vs. 21–26), a relatively larger tympanum (TD/HD 0.21–0.24 vs. 0.14–0.21), a higher maximal length of tail (205.0 vs. 174.0 mm), a higher maximal length of occipital spines (7.0 vs. 4.9 mm), a lower number of scales in the diastema between the nuchal and the dorsal crests (6–10 vs. 9–25), a larger mental (MW 1.4–1.9 vs. 1.0–1.5 mm, MH 1.2–2.0 vs. 1.1–1.7 mm). Most obvious is the difference in the color pattern: the black eye patch in *Acanthosaura tongbiguanensis* sp. nov. extends back and downwards beyond the posterior end of the tympanum, while it only extends to the anterior edge of the tympanum in *A. crucigera*; additionally, *Acanthosaura tongbiguanensis* sp. nov. has fewer or no spots on the dorsal surface of the body, whereas *A. crucigera* has more spots on the dorsal surface of the body.

*Acanthosaura tongbiguanensis* sp. nov. can be separated from *A. lepidogaster* based on its higher number of subdigital lamellae on the fourth finger (19–21 vs. 17–19) and the fourth toe (25–28 vs. 22–23), its bigger body size (93.0–115.6 vs. 76.5–101.1 mm), longer postorbital spines (3.6–6.3 vs. 1.5–2.5 mm, PS/HL 0.13–0.19 vs. 0.06–0.11) and longer occipital spines (4.5–7.0 vs. 3.2–3.4 mm, OS/HL 0.16–0.23 vs. 0.14–0.15), longer nuchal crest spines (4.0–6.7 vs. 2.9–3.4 mm, NSL/HL 0.15–0.21 vs. 0.12–0.15) and longer dorsal crest spines (2.4–4.2 vs. 1.5–2.7 mm, DS/HL 0.09–0.13 vs. 0.07–0.12), much higher maximal length of tail (205.0 vs. 144.1 mm), lower number of scales in the diastema between the nuchal and the dorsal crests (6–10 vs. 10–12), much wider rostral (3.3–4.5 vs. 2.8–3.0 mm), and larger gular pouch (1–2 vs. 0–1). The black eye patch in *Acanthosaura tongbiguanensis* sp. nov. extends backwards and downwards beyond the posterior end of the tympanum but never backwards and upwards to reach the black nuchal collar, while it usually does so in *A. lepidogaster*; the black nuchal collar extends downwards to reach the black oblique humeral fold, while it rarely reaches the black oblique humeral fold in *A. lepidogaster*; besides, the tongue and the inside of the mouth are pink in *Acanthosaura tongbiguanensis* sp. nov., while they are bluish-grey or black in *A. lepidogaster*; the postorbital spines, occipital spines, nuchal crest spines, the ridge of the rostralis, and orbit are lighter in color in *Acanthosaura tongbiguanensis* sp. nov., whereas they are darker in color in *A. lepidogaster*.

*Acanthosaura tongbiguanensis* sp. nov. can be separated from *A. murphyi* based on its smaller body size (93.0–115.6 vs. 103.7–127.3 mm) but relatively longer tail (TL/SVL 1.56–1.85 vs. 1.48–1.54), a higher number of subdigital lamellae on the fourth finger (19–21 vs. 15–18) and the fourth toe (25–28 vs. 21–23), shorter forelimb (34.7–43.2 vs. 49.8–56.6 mm) and hindlimb (54.1–63.9 vs. 60.4–68.4 mm), much shorter postorbital spines (3.6–6.3 vs. 5.6–11.8 mm, PS/HL 0.13–0.19 vs. 0.16–0.34), nuchal crest spines (4.0–6.7 vs. 7.0–14.9 mm, NSL/HL 0.15–0.21 vs. 0.24–0.43) and dorsal crest spines (2.4–4.2 vs. 2.6–10.5 mm, DS/HL 0.09–0.13 vs. 0.14–0.51), much lower width of the nuchal crest spines (1.0–1.5 vs. 2.9–4.8 mm), higher number of scales in the diastema between nuchal and dorsal crests (6–10 vs. 4–8), presence of occipital spines (vs. absence), a smaller gular pouch (1–2 vs. 4); the black eye patch in *Acanthosaura tongbiguanensis* sp. nov. extends backward and downward beyond the posterior end of the tympanum, while it usually extends backwards and upwards to reach the nuchal crests in *A. murphyi* (see species’ photographs in Nguyen et al. 2018).

*Acanthosaura tongbiguanensis* sp. nov. can be separated from *A. nataliae* by its smaller body size (93.0–115.6 vs. 106.7–158.0 mm) and a lower maximal tail length (205.0 vs. 287.0 mm) but a relatively longer tail (TL/SVL 1.56–1.85 vs. 1.2–1.5), much shorter length of postorbital spines (3.6–6.3 vs. 7.7–17.8 mm, PS/HL 0.13–0.19 vs. 0.36), nuchal crest spines (4.0–6.7 vs. 8.5–23.8 mm, NSL/HL 0.15–0.21 vs. 0.58) and dorsal crest spines (2.4–4.2 vs. 6.0–17.7 mm, DS/HL 0.09–0.13 vs. 0.44), a lower width of the nuchal crest spines (1.0–1.5 vs. 3.1–4.8 mm), lower width of mental (1.4–1.9 vs. 2.9 mm), a lower number of ventral scales (52–66 vs. 64–71), lower maximal length of forelimb (43.2 vs. 85.0 mm) and hindlimb (63.9 vs. 129.7 mm), presence of occipital spines (vs. absence), much lesser development of gular pouch (1–2 vs. 4), presence of light knee patch (vs. absence) and presence of a black nuchal collar (vs. absence); the black eye patch in *Acanthosaura tongbiguanensis* sp. nov. extends backward and downward beyond the posterior end of the tympanum but never continues backward to reach the black oblique folds anterior to the fore limb insertions while it usually does so in *A. nataliae* (see species’ description and photographs in Orlov et al. 2006).

*Acanthosaura tongbiguanensis* sp. nov. is distinguishable from *A. phongdienensis* based on its bigger body size (93.0–115.6 vs. 58.5–77.4 mm), longer tail (144.9–205.0 vs. 94.6–137.2 mm), higher number of subdigital lamellae on the fourth finger (19–21 vs. 14–17) and the fourth toe (25–28 vs. 19–23), longer postorbital spines (3.6–6.3 vs. 1.18–2.07 mm, PS/HL 0.13–0.19 vs. 0.06–0.09), longer nuchal crest spines (4.0–6.7 vs. 1.24–4.18 mm, NSL/HL 0.15–0.21 vs. 0.07–0.18) and longer dorsal crest spines (2.4–4.2 vs. 0.58–1.65 mm, DS/HL 0.09–0.13 vs. 0.03–0.07), a diastema between nuchal crests and dorsal crests (vs. a continuous nuchal and dorsal crest); the black eye patch in *Acanthosaura tongbiguanensis* sp. nov. extends backwards and downwards beyond the posterior end of the tympanum but never backwards and upwards to reach the black nuchal collar, while it does so in male *A. phongdienensis* (see species’ description and photographs in Nguyen et al. 2019), the postorbital spines, occipital spines, nuchal crest spines, the ridge of the rostralis and orbit are lighter in color in *Acanthosaura tongbiguanensis* sp. nov., whereas they are darker in color in *A. phongdienensis*.

*Acanthosaura tongbiguanensis* sp. nov. can be differentiated from *A. phuketensis* by having a higher number of subdigital lamellae on the fourth finger (19–21 vs. 15–17) and the fourth toe (25–28 vs. 21–24), a relatively longer tail (TL/SVL 1.56–1.85 vs. 1.4–1.7), much shorter postorbital spines (3.6–6.3 vs. 4.6–11.8 mm, PS/HL 0.13–0.19 vs. 0.23–0.38), nuchal crest spines (4.0–6.7 vs. 4.1–12.2 mm, NSL/HL 0.15–0.21 vs. 0.21–0.39) and dorsal crest spines (2.4–4.2 vs. 2.3–8.3 mm, DS/HL 0.09–0.13 vs. 0.11–0.26), a lower width of nuchal crest spines (1.0–1.5 vs. 1.4–2.9 mm), a lower maximal length of occipital spines (7.0 vs. 9.5 mm), a lower number of scales in the diastema between the nuchal and the dorsal crests (6–10 vs. 12–17), a bigger mental (MW 1.4–1.9 vs. 0.5–1.4 mm, MH 1.2–2.0 vs. 0.6–1.6 mm); the black eye patch in *Acanthosaura tongbiguanensis* sp. nov. never extends backward to reach the nuchal crest while it does so in male *A. phuketensis* (see species’ original description by Pauwels et al. 2015) and *Acanthosaura tongbiguanensis* sp. nov. has fewer or no spots on the dorsal surface of the body, whereas *A. phuketensis* has more spots on the dorsal surface of the body.

From *Acanthosaura titiwangsaensis*, the new species can be distinguished by its relatively larger tympanum (TD/HD 0.21–0.24 vs. 0.17–0.20), its longer tail (144.9–205.0 vs. 136.0–174.0mm, TL/SVL 1.56–1.85 vs. 1.1–1.5), higher maximal length of postorbital spines (6.3 vs. 4.4 mm) and nuchal crest spines (6.7 vs. 4.4 mm), higher length of dorsal crest spines (2.4–4.2 vs. 1.7–2.1 mm, DS/HL 0.09–0.13 vs. 0.07–0.09), much longer occipital spines (4.5–7.0 vs. 1.8–2.3 mm, OS/HL 0.16–0.23 vs. 0.09–0.10), lower number of scales in the diastema between the nuchal and the dorsal crests (6–10 vs. 10–13), presence of a light knee patch (vs. absence), less developed gular pouch (1–2 vs. 2–4), absence of medium-sized light orange spots edged in a faded black color on body and base of tail (vs. presence); the black eye patch in *Acanthosaura tongbiguanensis* sp. nov. extends backward and downward beyond the posterior end of the tympanum while it is restricted to the orbit and not extends into the postorbital region in *A. titiwangsaensis* (Wood et al. 2009).

**Figure 10.**
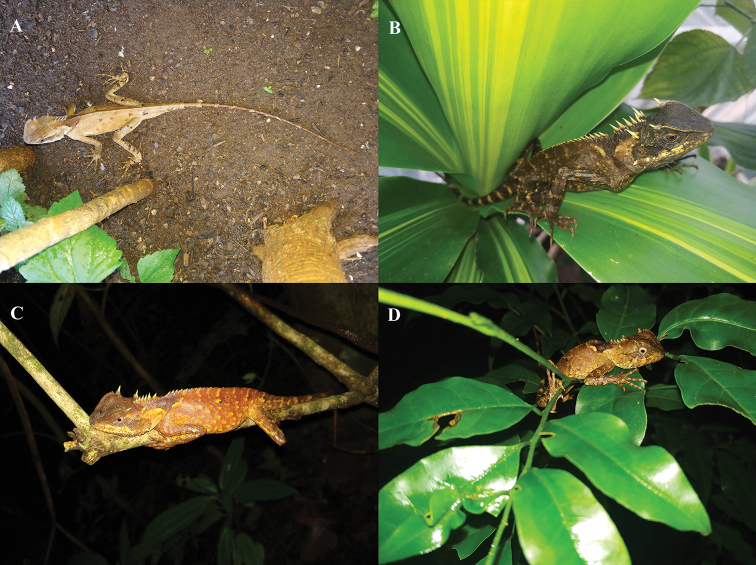
*Acanthosaura tongbiguanensis* sp. nov. **A** live adult male on the ground **B** live adult female on a leaf **C** live adult female asleep on a branch **D** live juvenile asleep on branches and leaves.

## Discussion

Although *Acanthosaura* collections from Myanmar and other Southeast Asian countries were not available for comparative analyses, we could demonstrate that *Acanthosaura tongbiguanensis* sp. nov. is a distinct species using data available from literature (Hardwicke and Gray 1827; Cuvier 1829; Günther 1861; Boulenger 1885; Orlovet al. 2006; Manthey 2008; Wood et al. 2009, 2010, Ananjeva et al. 2011; Grismer 2011; Pauwels et al. 2015; Nguyen et al. 2018; Nguyen et al. 2019).

Several morphometric characters of *Acanthosaura tongbiguanensis* sp. nov. overlap with some characters of other species in this genus, however, the new species can be differentiated from all other species of *Acanthosaura* by the black eye patch extending from the posterior margin of the nostrils through the orbit backwards and downwards to beyond the posterior end of the tympanum but neither meeting black nuchal collar nor the black oblique humeral fold (see Fig. 7).

The *Acanthosaura crucigera* group is wide ranging and its morphological variation is conserved, it is not surprising to find cryptic diversity within the *A. crucigera* complex (Wood et al. 2010). *Acanthosaura tongbiguanensis* sp. nov. was previously considered to represent *A. lepidogaster* (Yang et al. 2008) although it more closely resembles *A. crucigera*, however the numbers of subdigital lamellae on the fourth finger and toe of *Acanthosaura tongbiguanensis* sp. nov. are significantly different from *A. lepidogaster* and *A. crucigera*, and the molecular analyses also revealed them distinct taxa. Together with the species described herein *Acanthosaura* currently comprises 14 species in total.

## Supplementary Material

XML Treatment for Acanthosaura tongbiguanensis
